# Validation of the Arabic version of the cyberchondria severity scale 12 items (CSS-12-Ar) among a sample of Lebanese adults

**DOI:** 10.1186/s12888-023-05123-x

**Published:** 2023-08-23

**Authors:** Souheil Hallit, Radosław Rogoza, Carl Abi Semaan, Vanessa Azzi, Toni Sawma, Sahar Obeid

**Affiliations:** 1 School of Medicine and Medical Sciences, Holy Spirit University of Kaslik, P.O. Box 446, Jounieh, Lebanon; 2 Research Department, Psychiatric Hospital of the Cross, Jal Eddib, Lebanon; 3 Applied Science Research Center, Applied Science Private University, Amman, Jordan; 4 University of Economics and Human Sciences in Warsaw, Warsaw, Poland; 5 Social Innovation Chair, University of Lleida, Lleida, Spain; 6 Social and Education Sciences Department, School of Arts and Sciences, Lebanese American University, Jbeil, Lebanon

**Keywords:** Cyberchondria severity, CSS-12, Arabic, Psychometric properties, Validation, Lebanon

## Abstract

**Background:**

To the best of our knowledge, the Cyberchondria Severity Scale-12 (CSS-12) has not been translated into Arabic; therefore, our objective was to assess the psychometric properties of the Arabic version of the CSS (CSS-12-Ar) among a sample of Lebanese adults.

**Methods:**

Participants were enrolled in January 2021. A confirmatory factor analysis (CFA) was carried out using the MPlus software v.7.2, reporting several goodness-of-fit indicators: Relative Chi-square (χ2/df), Root Mean Square Error of Approximation (RMSEA), Comparative Fit Index (CFI) and Tucker Lewis Index (TLI). To evaluate measurement invariance across gender, we conducted higher-order multiple group confirmatory analysis using lavaan software.

**Results:**

449 participants enrolled in this study (mean age: 24.34 ± 8.22 years, 70.6% females). Since the correlations between the four-factor model were very high (r > 0.8), we ran the higher-order CFA in which all first-order latent variables were loading a general factor. The analyzed model was well-fitted to the data χ^2^_(50)_ = 173.34; *p* < 0.001; CFI = 0.926; RMSEA = 0.074 [0.062, 0.086]. The Cronbach’s alpha values were good for the total score (0.92), as well as for excessiveness (0.80), distress (0.77), reassurance (0.81) and compulsion (0.76). The results provided evidence of full scalar invariance across gender. The comparison of latent mean scores revealed no significant differences across gender, in either the cyberchondria total score or its facets. The CSS-12 score was positively associated with anxiety (*r* = 0.10; *p* = 0.003) (convergent validity), OCD (*r* = 0.11; *p* = 0.016) and stress (*r* = 0.35; *p* < 0.001) (concurrent validity).

**Conclusion:**

The CSS-12-Ar was deemed a suitable scale to measure the severity of cyberchondria among Lebanese university students. We hope that researchers and clinicians can benefit now from this scale.

**Supplementary Information:**

The online version contains supplementary material available at 10.1186/s12888-023-05123-x.

## Background

With the increasing impact of digital technology, subjects using the Internet as a source for health information is steadily increasing. In terms of prevalence, it is estimated that 62% of American adults search for health information online; thus constituting the fifth most common online activity [1]. The numbers in Saudi Arabia were even higher than those in the United States, with 88% of the participants in a study searching for health information [2]. In terms of efficiency, different studies have focused on the benefits of searching online for health information [3, 4], among others the ease of access to information, anonymity, the search process is cost-effective, patient empowerment and support provided in the context of informed decision-making [5, 6]. However, the majority of studies have focused on the misdeeds of this research. For example, a previous study conducted by Lauckner and Hsieh (2013) [7] focused on negative emotional responses related to seeking health information online. Other studies have also highlighted the association between this type of research and the escalation of health anxiety [8], as well as the increased risk of developing anxiety disorders [9]. Recently, researchers coined the term “cyberchondria” to describe this process of heightened anxiety about physical health due to excessive searching for health information online [10]. Cyberchondria is defined as “an excessive or repeated research for health-related information on the Internet, driven by distress or anxiety about health, which amplifies such distress or anxiety” [10]. In this context, cyberchondria combines a behavioral pattern (i.e., over-researching the web) with a subsequent emotional state (i.e., excessive concern about health). Thus, the main feature of cyberchondria is the escalation/excessiveness element, whereby subjects spend excessive and increasing amount of time searching for information [11, 12].

To assess cyberchondria, McElroy and Shevlin developed the Cyberchondria Severity Scale (CSS-33 items) on a sample of 190 undergraduates [13]. The CSS was shown to have satisfactory psychometric qualities and high internal reliability (Cronbach alphas ranging from 0.75 to 0.95). It has also been demonstrated to have strong concurrent and convergent validity (13). Despite its adequate psychometric properties, recommendations to refine the CSS were suggested by removing the “Mistrust” factor due to its theoretical ambiguity, its weak correlation with the other factors of the scale and its failure to load on the total cyberchondria score [14]. The validation was reproduced in a community sample by Norr and colleagues [15]. Their findings indicated that an orthogonal general factor with four subfactors provides the greatest model fit and that mistrust of medical professionals does not belong to the cyberchondria construct. In terms of contemporaneous validity, the CSS was found to have favorable relationships with health anxiety [15, 16] and the Depression, Anxiety, and Stress Scale [13]. Furthermore, the scale has been criticized for its length and the inclusion of several items that may not be relevant or specific to cyberchondria [12, 17]. Barke et al. created a shortened version of the CSS (CSS-15 items) [17], but it showed a lower internal consistency the total and subscales scores than the original scale. In a second attempt, twelve items were chosen to be retained based on the exploratory factor analysis, creating a brief, reliable, and valid measure of cyberchondria (CSS-12), divided into four previously identified factors: Compulsion, Distress, Excessiveness, and Reassurance. Confirmatory bifactor modeling showed that the CSS-12 is best scored as a unidimensional scale, although the subscales may provide useful additional information [18].

The CSS is validated in multiple languages, including but not limited to Italian [19], Turkish [20], German [17], Polish [21], Brazilian Portuguese [22], and Russian [23]. Scales’ translation to a different language might result in non-equivalent measures especially in science/health since the language and culture might influence those outcomes [24]. In 2018, the world economic forum listed online misinformation as one of the top ten threats to humans [25]. Research has emphasized that misinformation can lead to health anxiety [26], poor health outcomes [27], and impairment leading to inability to estimate the severity of ongoing problems [28]. Lebanon, a country suffering not only the consequences of the COVID-19 pandemic, but also of a devastating explosion and economic, social, and political crises. Therefore, it is ever more important to seek ways to improve the physical and mental health of a population experiencing great adversity. However, to be able to intervene in a specific and successful way at various levels, it is it is important to validate a tool for health care professionals living in the Arabic speaking countries and specifically in Lebanon to assess cyberchondria. Since the syndrome is motivated by concern about prospective health disorders or symptoms, the perceived severity of a specific circumstance may vary depending on cultural background and the current country situation. Both individual characteristics and contextual circumstances naturally influence assessment and coping strategies. Stressor evaluations are generally associated with higher overall levels of stress and its mental health correlates, such as depression and anxiety, as being more severe or frightening [29]. In addition, to the best of our knowledge, the CSS scale has not been translated into Arabic, therefore, our objective was to assess the psychometric properties of the Arabic version of the CSS (CSS-12-Ar) in terms of number of factors and internal consistency, as well as its validity among a sample of Lebanese adults. We expect that the scale will show four factors (H1), will have a good internal consistency (H2), and will be positively correlated with mental health issues (obsessive-compulsive disorder, anxiety and stress) (H3); in fact, positive associations have been stated between cyberchondria and health anxiety [14, 15], depression, anxiety and stress [13].

## Methods

### Participants

A total of 449 adults agreed to enroll in this study. The mean age of the sample was 24.34 ± 8.22 years, with 70.6% females. The mean cyberchondria severity score was 15.91 ± 9.64. Table 1 includes more details about the sample.

**Table 1:** Sociodemographic and other characteristics of the participants (N = 449)

Variable	N (%)
Gender	
Male	132 (29.4%)
Female	317 (70.6%)
Marital status	
Single/widowed/divorced	364 (81.1%)
Married	85 (18.9%)
Education level	
Complementary or less	34 (7.6%)
Secondary	61 (13.6%)
University	354 (78.8%)
	**Mean ± SD**
Age (in years)	24.34 ± 8.22

### Study design

This is a cross-sectional study that was conducted during the COVID-19 pandemic (January 2021) through an online questionnaire. The total amount of participants was 449 individuals. A snowball sampling method was used to collect participants from all Lebanese governorates (Beirut, Bekaa, Mount Lebanon, South Lebanon and North Lebanon). The survey was completely anonymous and was distributed via the Whatsapp social application. Individuals above the age of 18 and residents of Lebanon were included [30].

### Minimal sample size calculation

A minimal sample of 240 participants was deemed necessary to validate the CSS-12 scale, based on 20 participants per 1 scale item [31].

### Questionnaire

The language of the questionnaire was Arabic, the official language of Lebanon. The questionnaire consisted of a sociodemographic section and different scales:

#### Sociodemographic section

Information about age, educational level, marital status, number of children, household crowding index (according to the number of rooms in the house excluding bathroom(s) and kitchen relative to the number of residents [32]) was collected.

#### Cyberchondria severity scale (CSS)

The CSS in a brief 12-item version assessed compulsion, distress, excessiveness and reassurance, without the mistrust of medical professional dimension [33]. The forward and backward translation method was applied to the CSS-12 scale following international guidelines [34]. It was translated from English to Arabic by a clinical psychologist, which was verified by a professional medical writer. Another clinical psychologist who is fluent in Arabic backward translated the scale from Arabic to English (Appendix 1). The backward-translated questionnaire was matched by the study’s principal investigator in order to detect inconsistencies and solve discrepancies between the two versions. A pilot study was conducted on 30 persons before the start of the official data collection to make sure all questions are well understood; no changes were done consequently.

The CSS-12 items are scored on a Likert-type scale ranging from “1 = never” to “5 = always”. Higher scores reflected higher cyberchondria. Four factors derive from this scale: Excessiveness, distress, reassurance and compulsion. The Cronbach’s alpha values for the four subscales in this study were as follows: 0.80, 0.77, 0.81 and 0.76 respectively. Permission to use the scale was obtained from Dr Eoin McElroy.

We used the Lebanese Anxiety Scale (LAS-10) to assess the CSS-12 convergent validity since spending more time on the internet searching for health information (i.e. cyberchondria) is associated with higher anxiety according to previous findings [25]. The LAS-10 evaluates anxiety among Lebanese adults [35] and adolescents [36]. It consists of 1- items rated differently: items 1 to 7 are scored from “0 = not present” to “4 = very severe” and items 8 to 10 are scored from “1 = never or almost never” to “4 = almost always” [35]. Higher scores reflect higher anxiety The Cronbach’s alpha values in the original study was 0.86 and 0.93 in the current one.

#### Beirut distress scale

The BDS-10 [37] assesses the level of distress among Lebanese adults. Items are rated from “0 = not at all” to “3 = all of the times”. Higher scores indicate higher levels of stress. The Cronbach’s alpha values in the original and current study were 0.90 and 0.89 respectively.

#### Yale brown obsession compulsion scale (YBOCS)

The YBOCS assesses the severity of OCD. It includes 10 items rated from 0 to 4 indicating no symptoms to extreme symptoms. Higher scores reflected more severe obsessive-compulsive symptoms [5]. The Arabic version of this scale has been previously used in a previous project [38]. The Cronbach’s alpha values in the original and current study were 0.89 and 0.86 respectively.

We used the YBOCS and BDS-10 scales to assess divergent validity, since previous research emphasized the manifestation of obsessive-compulsive components in cyberchondria [16, 39] and since higher distress may be one of the features associated with a form of behavioral addiction [40].

### Statistical analysis

No missing data was found in the database as all questions were required in the Google form. The Mplus v. 7.2 was used to conduct confirmatory factor analyses (CFA) of the CSS-12. Multiple indices of goodness-of-fit were described: the Root Mean Square Error of Approximation (RMSEA) (close fit are considered for values < 0.08) and the Comparative Fit Index (CFI) (acceptable values are ≥ 0.90) [41]. We used maximum likelihood estimation with robust means and standard errors. evidence of convergent validity was assessed in this subsample using the average variance extracted (AVE), with values of ≥ 0.50 considered adequate [42]. To evaluate measurement invariance across gender, we conducted higher-order multiple group confirmatory analysis. The analysis was carried out in lavaan [43]. Three models were assessed: configural (i.e., without constraints); metric (i.e., with constraint factor loadings), and scalar (i.e., with constraint item intercepts). The model was considered as invariant at configural level if RMSEA and CFI values were acceptable as outlined above. The model was considered invariant at the metric and scalar levels if the difference in subsequent models did not exceeded 0.015 in RMSEA and 0.010 in CFI [44].

The statistical analysis was conducted on the SPSS software version 25. Reliability was checked using Cronbach’s alpha for all scales and subscales. The sample was normally distributed as verified by the skewness and kurtosis of the CSS score, which varied between − 1 and + 1 [45]. These conditions consolidate the assumptions of normality in samples larger than 300 [46]. Pearson correlation test was used to correlate two continuous variables. The Student t test was used to compare the cyberchondria score between genders. Significance was set at a p < 0.05.

## Results

### Factor validity- hypothesis 1

**Fig. 1:**
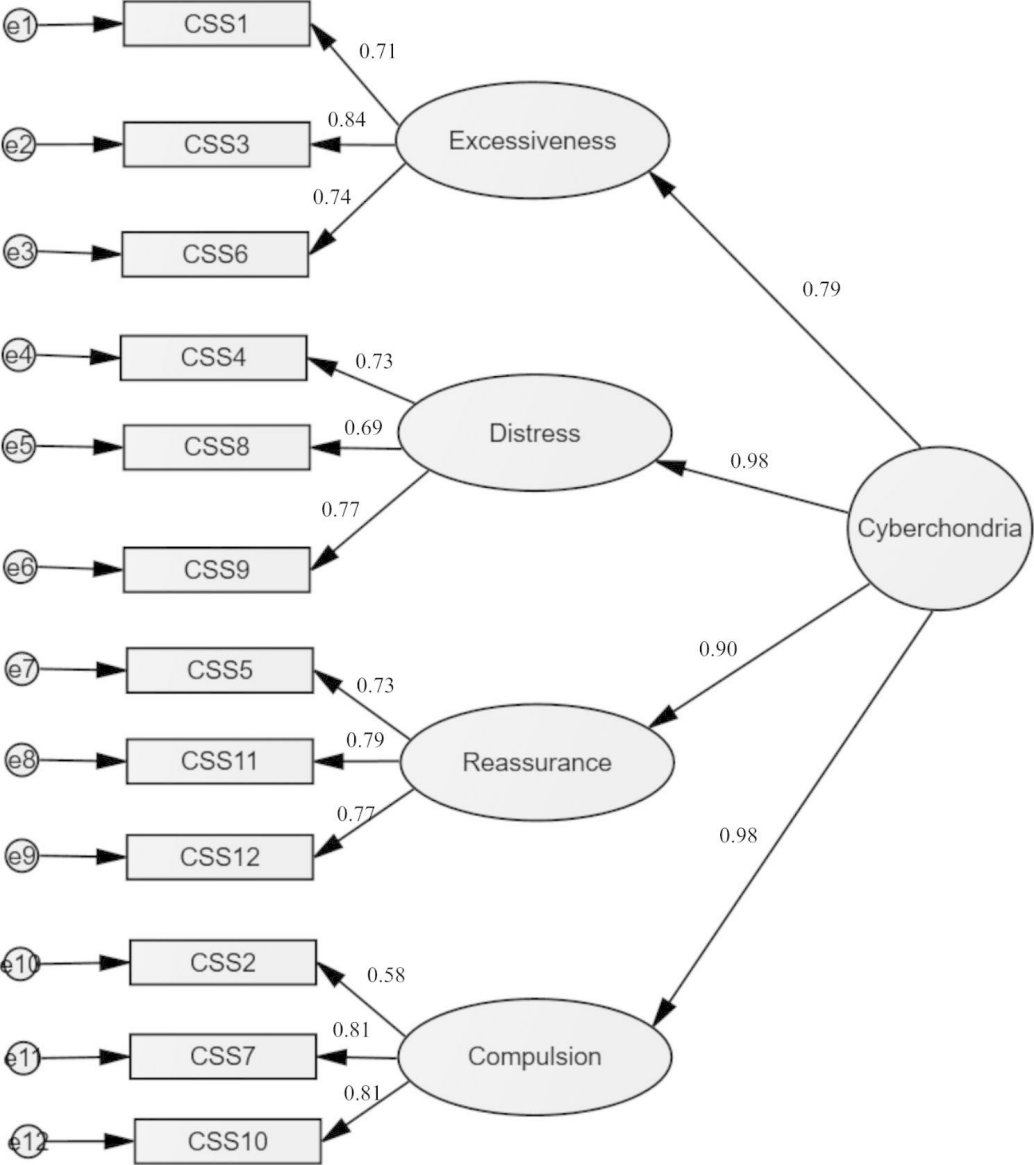
Standardized factor loadings of the four-factor model of the Arabic version of the Cyberchondria Severity Scale (CSS-12-Ar).

Since the correlations between the four-factor model were very high (r > 0.8), we ran the higher-order CFA in which all first-order latent variables were loading a general factor. The analyzed model was well-fitted to the data χ^2^_(50)_ = 173.34; *p* < 0.001; CFI = 0.926; RMSEA = 0.074 [0.062, 0.086]. Standardized factor loadings of this model are summarized in Fig. 1. The AVE was adequate = 0.51.

### Internal consistency- hypothesis 2

The Cronbach’s alpha values were good for the total score (0.92), as well as for excessiveness (0.80), distress (0.77), reassurance (0.81) and compulsion (0.76). The total item correlations varied between 0.63 and 0.78 (Table 2).

**Table 2:** Correlation between the total score and each item of the scale

Item	Total-item correlation
CSS 1	0.63
CSS 2	0.65
CSS 3	0.75
CSS 4	0.75
CSS 5	0.74
CSS 6	0.77
CSS 7	0.78
CSS 8	0.70
CSS 9	0.76
CSS 10	0.77
CSS 11	0.73
CSS 12	0.72

Numbers refer to Pearson correlation coefficients; p < 0.001 for all correlations.

### Convergent and concurrent validity- hypothesis 3

The CSS-12 score was positively associated with anxiety (*r* = 0.10; *p* = 0.003) (convergent validity), OCD (*r* = 0.11; *p* = 0.016) and stress (*r* = 0.35; *p* < 0.001) (concurrent validity).

### Comparison between gender

The results of the measurement invariance testing are presented in Table 3. The results provided evidence of full scalar invariance across gender. The comparison of latent mean scores revealed no significant differences across gender, in either the cyberchondria total score or its facets. Similarly, we also observed no significant differences when analyzing mean scores of cyberchondria total score between females (*M* = 16.21, *SD* = 9.89) and males (*M* = 15.20, *SD* = 9.01) in the total sample, *t*(447) = -1.01, *p* = 0.315.

**Table 3:** Measurement Invariance across Gender

Model	χ^2^_(df)_	*p*	CFI	RMSEA	ΔCFI	ΔRMSEA
Configural	241.04_(100)_	< 0.001	0.919	0.079	-	-
Metric	257.82_(111)_	< 0.001	0.916	0.077	0.003	0.002
Scalar	269.48_(118)_	< 0.001	0.913	0.076	0.003	0.001

## Discussion

To the best of our knowledge, the Arabic version is the first validation of the CSS-12 in a language other than English. In terms of dimension assessment, the higher-order factor model showed acceptable fit indices. Although the original version showed four factors, the latent correlations between those factors turned out to be very high in our study (r > 0.8), suggesting that these factors almost measure the same component, with limited utility in practice in differentiating them from each other. Previous research also encountered such heightened latent correlations and attempted to account for them through the introduction of a bifactor [18]. Such approach, however, seldom solves the problem as the bifactor is most frequently accounting for noise variance representing implausible response patterns [47]. Furthermore, bifactor model frequently provides better fit to the data even if an a priori non-bifactor structure is known [48]. Thus, the proposed higher-order model highlights that all of the four factors have some psychological meaning, therefore, the scale could be interpreted in terms of a general score. Thus, the first hypothesis was verified.

The internal consistency of the CSS-12-Ar was very good for the total scale and each factor alone. The alpha value was higher in the Arabic version for the total scale and factor 4, but lower for Factors 1, 2 and 3 compared to the English version [33]. All values were above the commonly suggested threshold of 0.70 [49, 50]. In addition, the total-item correlations were moderate to large, therefore, we can consider that we verified the second hypothesis as well.

The CSS-12-Ar total score was positively correlated with anxiety, in line with previous findings [33]. Cyberchondria is by definition an anxiety resulting from online search about health information [11, 12]; it was strongly associated with health anxiety in the original paper (r = 0.53) but moderately with anxiety in general (r = 0.3) as measured by the Generalized Anxiety Disorder scale (GAD-7) [33]. Our results showed that it was weakly associated with the LAS score, making our results seem logical and expected. Furthermore, our results showed that higher cyberchondria was significantly associated with more distress and OCD, in line with previous research [51, 52]. We need to note that this study was conducted during the COVID-19 pandemic, a period that is associated with more mental health issues [53]. Consequently, the third hypothesis regarding both convergent and divergent validity was also verified.

### Clinical implications

With the increasing use of the internet in Lebanon as in the world, access to health information is gradually increasing; therefore, it has become compelling to be aware of the potential health threats of cyberchondria and its evaluation tools. As a result, the current findings will help researchers develop measures to mitigate the harmful effects of cyberchondria, implement policies, and raise awareness about cyberchondria, particularly among at-risk individuals. As a result, future efforts may concentrate on improving systems for effective monitoring of internal discomfort and increasing ways to assist individuals in gaining a sense of personal control.

### Limitations

Symptoms were self-report (not evaluated by a healthcare professional) and thus are subjective. In addition, recall bias might be present, which may have led to an overestimation of the answers given to some questions. Participants may have misunderstood some of the questions, which is a source of information bias. Additionally, a main reason for selection bias is the refusal rate and the fact that the sample was recruited via the snowball technique. In addition, most participants were females, single, with a university level of education, which limits the generalizability of the findings. Confounding bias might be present since some information about participants’ occupation and mental health disorders were not assessed. Content validity of the instrument and test–retest reliability of scale were not conducted. Research on different study groups (e.g., clinical or community-based sample) and taking this study limitations into consideration, are needed.

## Conclusion

Our goal in this study was to provide evidence of validity of the CSS-12-Ar, which was done by inspection of its factor, convergent, and divergent validity as well as evaluating its internal consistency. All postulated hypotheses were verified by our database. Consequently, researchers and clinicians can take advantage of the CSS-12-Ar to assess cyberchondria levels in Lebanon.

### Electronic supplementary material

Below is the link to the electronic supplementary material.

Supplementary Material 1: Cyberchondria Severity Scale (CSS-12-Ar)

## Data Availability

All data generated or analyzed during this study are not publicly available due the restrictions from the ethics committee (data are owned by a third-party organization). The dataset supporting the conclusions is available upon request to the corresponding author (SH).
