# Supplementary material for: Validation of the Arabic version of the cyberchondria severity scale 12 items (CSS-12-Ar) among a sample of Lebanese adults
Source: BMC Psychiatry. 2023 Aug 23;23:618. doi: 10.1186/s12888-023-05123-x (PMC10463298; doi:10.1186/s12888-023-05123-x)
Supplement: Supplementary file 1 — Supplementary Material 1: Cyberchondria Severity Scale (CSS-12-Ar) [file 12888_2023_5123_MOESM1_ESM.docx]

Cyberchondria Severity Scale (CSS-12-Ar)

|  | Never  أبداً | Rarely  نادراً | Sometimes  أحياناً | Often  غالباً | Always  دائماً |
| --- | --- | --- | --- | --- | --- |
| 1. If I notice an unexplained bodily sensation I   will search for it on the internet  إذا لاحظت إحساسًا جسديًا غير مبرر سأبحث عنه على الإنترنت |  |  |  |  |  |
| 1. Researching symptoms or perceived medical   conditions online distract me from reading news/sports/entertainment articles online  البحث عن الأعراض أو الحالات الطبية المتصورة على الإنترنت يصرف إنتباهي عن قراءة الأخبار/ الرياضة/ المقالات الترفيهية على الإنترنت |  |  |  |  |  |
| 1. I read different web pages about the same   perceived condition  قرأت صفحات ويب مختلفة عن نفس الحالة المتصوّرة |  |  |  |  |  |
| 1. I start to panic when I read online that a   symptom I have is found in a rare/serious condition  بدأت أشعر بالذعر عندما قرأت على الإنترنت أن أحد الأعراض التي أعاني منها وجدت في حالة نادرة / خطيرة |  |  |  |  |  |
| 1. Researching symptoms or perceived medical   conditions online lead me to consult with my GP  يقودني البحث عن الأعراض أو الحالات الطبية المتصورة عبر الإنترنت إلى التشاور مع طبيبي العام |  |  |  |  |  |
| 1. I enter the same symptoms into a web search   on more than one occasion  أدخل نفس الأعراض في بحث الويب في أكثر من مناسبة |  |  |  |  |  |
| 1. Researching symptoms or perceived medical   conditions online interrupt my work (e.g. writing emails, working on word documents or spreadsheets)  يؤدي البحث عن الأعراض أو الحالات الطبية المتصورة عبر الإنترنت إلى مقاطعة عملي (مثل كتابة رسائل البريد الإلكتروني أو العمل على مستندات Word أو جداول البيانات) |  |  |  |  |  |
| 1. I think I am fine until I read about a serious   condition online  أعتقد أنني بخير حتى قرأت عن حالة خطيرة على الإنترنت |  |  |  |  |  |
| 1. I feel more anxious or distressed after   researching symptoms or perceived medical conditions online  أشعر بمزيد من القلق أو الحزن بعد البحث عن الأعراض أو الحالات الطبية المتصورة عبر الإنترنت |  |  |  |  |  |
| 1. Researching symptoms or perceived medical   conditions online interrupt my offline social activities (e.g., reduces time spent with friends/family)  يؤدي البحث عن الأعراض أو الحالات الطبية المتصوّرة عبر الإنترنت إلى مقاطعة أنشطتي الاجتماعية غير المتّصلة بالإنترنت (على سبيل المثال، تقليل الوقت الذي أقضيه مع الأصدقاء / العائلة) |  |  |  |  |  |
| 1. I suggest to my GP/medical professional that I may need a diagnostic procedure that I read about online (e.g., a biopsy/ a specific blood test)   أقترح على طبيبي العام أنني قد أحتاج إلى إجراء تشخيصي قرأت عنه عبر الإنترنت (على سبيل المثال، خزعة / اختبار دم محدد) |  |  |  |  |  |
| 1. Researching symptoms or perceived medical   conditions online lead me to consult with other medical specialists (e.g., consultants)  البحث عن الأعراض أو الحالات الطبية المتصوّرة عبر الإنترنت يقودني للتشاور مع الأطباء الأخصائيين الآخرين (مثل الاستشاريين) |  |  |  |  |  |
